# Aortic Cannulation around Grade 5 Aortic Arch Atheroma Utilizing Transesophageal Echocardiography

**DOI:** 10.1155/2020/8820948

**Published:** 2020-11-05

**Authors:** Trey W. Vanek, Jeremiah Hayanga, Matthew Ellison, Jeffrey Puette, Lawrence Wei, Heather K. Hayanga

**Affiliations:** ^1^Department of Anesthesiology, West Virginia University, Morgantown, WV, USA; ^2^Department of Cardiovascular and Thoracic Surgery, West Virginia University, Morgantown, WV, USA; ^3^Division of Cardiovascular and Thoracic Anesthesiology, Department of Anesthesiology, West Virginia University, Morgantown, WV, USA

## Abstract

A 61-year-old male with severe aortic valve stenosis was scheduled for a minimally invasive bioprosthetic aortic valve replacement. Intraoperative transesophageal echocardiography (TEE) showed a unicuspid aortic valve and extensive aortic atheromatous disease. A large atheroma with mobile components existed near the distal aortic arch. A 17-French aortic cannula was successfully placed using TEE guidance with the tip proximal to the mobile atheroma to avoid inadvertent disruption and subsequent embolic sequelae. The patient had no evidence of perioperative stroke or other complications postoperatively. This case demonstrates one strategy to manage severe atheromatous disease intraoperatively. We also review additional management options.

## 1. Introduction

Cardiopulmonary bypass requires venous and arterial cannulation. The arterial cannula is most commonly inserted into the ascending aorta to return oxygenated blood to the patient's circulation [1]. Atherosclerosis of the ascending aorta and aortic arch is important to identify before cannulation because manipulation of a diseased aorta increases the risk of intraoperative complications such as stroke and other embolic phenomena. Particulate emboli originating from the aortic atheroma have been shown to cause neurological complications postoperatively as well [2]. In fact, the extent of the ascending aorta atheroma is tracked as a variable in the Society of Thoracic Surgeons database [3]. Depending on the imaging studies obtained, preoperative imaging may identify significant aortic atherosclerosis. Often though, the extent of atheromatous disease may not be appreciated until intraoperative evaluation. Various intraoperative imaging modalities can be used to diagnose significant aortic atheromatous disease and then subsequently guide the surgical approach.

After detection and visualization of the location of the patient's pathology, the surgical team can reassess the intended strategy for cardiopulmonary bypass and tailor the approach to the individual. A study by Trehan et al. showed that identifying and then, as a result, altering the surgical technique of patients with mobile aortic atheromas corresponded to a low perioperative stroke rate of 0.96% [4]. By taking certain considerations into mind, it is possible to reduce perioperative complications related to aortic atherosclerosis. HIPAA authorization was obtained from the patient for the publication of this report.

## 2. Case Description

**Figure 1:**
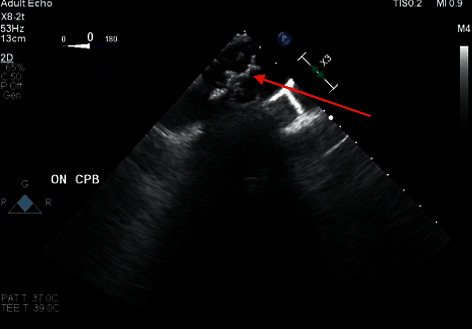
Upper esophageal aortic arch in long axis showing grade 5 aortic arch atheroma.

A 61-year-old male with a past medical history of hypertension, hyperlipidemia, chronic obstructive pulmonary disease, valvular cardiomyopathy, substance abuse, and coronary artery disease presented to the emergency department with progressively worsening shortness of breath. The patient had associated weakness and fatigue in the days leading up to his hospital admission. The patient's cardiac enzymes were also elevated. Cardiac catheterization was significant for 50% stenosis of the proximal and mid-left anterior descending artery. Transthoracic echocardiography (TEE) showed severe aortic valve stenosis with moderate insufficiency and an ejection fraction of 32%. Subsequently, he was scheduled for a minimally invasive aortic valve replacement with a 27 mm·St. Jude Trifecta stented pericardial bioprosthesis under general endotracheal anesthesia. Standard monitors were used including cerebral oximetry. Intraoperative TEE showed a significant atherosclerotic burden with grade 3 atheroma in the ascending aorta and grade 5 atheroma in the descending aorta and aortic arch [5] (Figure 1). In the distal aortic arch, there was a large complex atheroma with a mobile component. The aortic valve was unicuspid, heavily calcified, and severely stenosed. Prior to initiating cardiopulmonary bypass, the aorta was palpated, and areas of calcification were noted. With good visualization of the discrete large complex atheroma using TEE, it was felt that modifying the cardiopulmonary bypass approach or using epiaortic ultrasound would yield no further potential benefit. A purse-string suture was placed in an area that was free of calcium. A 17-French Bio-Medicus aortic cannula was meticulously placed using the Seldinger technique and TEE guidance to direct placement of the tip away from the mobile atheroma that was visualized in the aortic arch. First described in 2006 for aortic cannulation, the Seldinger method is helpful when cannulating a patient with thoracoabdominal aortic disease, including a calcified ascending aorta or complicated aortic dissection [6]. A retrograde coronary sinus perfusion cannula was then placed. Aortic root venting and cardioplegia catheters were placed. The aorta was cross-clamped in the area with least disease as determined by manual palpation and TEE guidance. The aortic valve replacement was completed with no complications. The postoperative TEE showed that the previously described aortic arch atheroma remained intact. The patient tolerated the operation well and had no perioperative stroke events or other complications. He was discharged home in stable condition five days postoperatively.

## 3. Discussion

Detecting cardiac surgery patients at risk for significant complications related to aortic atheromatous disease is paramount. The use of TEE has been recommended for all cardiac and thoracic aortic surgeries rather than only in selected complex operations [7]. A comprehensive TEE examination at the start of surgery before sternotomy is used to screen for atherosclerosis of the aorta [8]. If significant atherosclerosis is visualized, additional imaging may be prudent to further assess the diseased areas and potentially alter the surgical approach. A meta-analysis showed that the sensitivity and specificity for the use of TEE for the detection of aortic arch atherosclerosis were 21% and 99%, respectively [9]. Therefore, atherosclerosis can be considered present with a positive result, while the absence of atheroma on TEE does not necessarily rule out atherosclerosis being present. If suspicious, further evaluation may be performed using modified TEE views or with epiaortic ultrasound, which is thought to be a superior technique to TEE for the detection and localization of atherosclerosis [10]. Epiaortic ultrasound has the advantage of more focal visualization of both the proximal and distal ascending aorta.

When comparing TEE, epiaortic ultrasound, and CT for imaging atherosclerosis, TEE has the advantage of being performed before incision and in real time during cannula placement. Although all three imaging modalities can provide valuable information, the choice depends on operator experience and availability [11]. TEE was chosen in this case for a number of reasons. Our patient had computed tomography of the chest without intravenous contrast preoperatively to evaluate for aortic calcification. In the ascending aorta, this was only significant for mild calcified atherosclerosis at the posterior aspect of the aorta. The anesthesiologist and surgeon were confident that the soft-tip guidewire could be visualized at all times using TEE during placement of the wire and cannula. Epiaortic ultrasound was not performed because the quality of the TEE images obtained and the location of the pathology made steering the aortic cannula away from the discrete pathology using TEE guidance adequate.

After detection, the patient care team must develop an anesthetic and surgical plan going forward. The first consideration should be to determine the ideal site for arterial cannulation. In cases of localized atherosclerosis, the ascending aorta can still be used as the cannulation site by directing the tip of the cannula away from the atheroma with TEE visualization as demonstrated in this case. In cases of more diffuse or severe atherosclerosis, alternate sites for arterial cannulation, such as the axillary artery, femoral artery, or subclavian artery, may be warranted [8]. The choice of the arterial cannulation site should depend on the proposed procedure, operative strategy, and extent of atherosclerosis or other aortic pathology. In patients with severe atherosclerosis of the aorta, the right axillary artery is a preferred arterial inflow site [12]. A recent study showed that right axillary artery cannulation provides sufficient antegrade flow for cardiopulmonary bypass [13]. Of the 76 patients who were involved in the study, 28 of them had severe aortic atherosclerosis as the indication for cannulation of the right axillary artery. Permanent perioperative strokes were observed in 2 of the patients; however, these events did not occur during the operation, and no other embolic events were observed in any of the other patients. In our case, the surgical and anesthesia teams decided to proceed with cannulation of the ascending aorta due to the fact that they were able to clearly visualize the large complex atheroma using TEE. Furthermore, although the Seldinger technique poses a hypothetical risk of aortic arch atheroma disruption during cannulation, the literature describing the incidence of such a complication is lacking. In our case, we were confident that we maintained visualization of the tip of the wire using TEE.

Other management strategies include taking into consideration arterial cannula type, using cerebral oximetry, and recognizing the potential effects of cross-clamping. Multiple different designs of arterial cannulae exist, and it is up to the surgical team to tailor their choice to the situation [14]. Bent-tip cannulae have been designed to reduce the shear stress on the aortic wall caused by a straight cannula, which may direct high-velocity blood flow at the wall. Low-velocity cannulae are also available. As for anesthetic management, a large retrospective study of 1698 patients showed a significant reduction in the perioperative stroke rate when cerebral oximetry was used to optimize and maintain intraoperative cerebral oxygenation [15]. Cross-clamping of the aorta is also a major risk factor for perioperative stroke during cardiac surgery [16]. Models have shown that cross-clamping of the aorta produces a substantial output of particulate matter [17]. Patients with atherosclerotic areas of their aorta, therefore, are at an even greater risk. Alternatives to cross-clamping may include altering the proposed location of clamping based on TEE or palpation of the aorta, as done in this case; altering the location based on epiaortic ultrasound; endoballoon occlusion; or deep hypothermic circulatory arrest. However, neither endoballoon occlusion nor deep hypothermic circulatory arrest has been proven to reduce the risk of perioperative embolic events [8].

In summary, a variety of management strategies may be used to diagnose and manage patients with significant aortic atherosclerosis requiring cardiopulmonary bypass. The strategy in this case involved using TEE to identify the pathology and then to visualize aortic cannulation to guide the cannula away from the large mobile atheroma. This method worked to successfully place the patient on cardiopulmonary bypass without atheroma dislodgement or clinically significant embolization from other areas of the diseased aorta. Other diagnostic modalities include modified TEE and epiaortic ultrasound. Management considerations include changing the location of arterial cannulation, selecting different types of cannulae such as bent tip or low-velocity, using cerebral oximetry, and being cognizant of the risks of cross-clamping a diseased aorta. The approach that is implemented should be considered on a case-by-case basis. Through being vigilant with diagnosis and considering various strategies to address significant aortic atherosclerosis in patients requiring cardiopulmonary bypass, the ultimate goal is to reduce perioperative and postoperative complications.

## Data Availability

The research data used to support the content of this case report are cited within the article with the list of references provided.

## Glossary

Transesophageal echocardiography: TEE.
